# Evaluation of the Effect of Body Mass Index and Waist Circumference on Ocular Health Parameters in Children and Adolescents

**DOI:** 10.3390/children12040413

**Published:** 2025-03-26

**Authors:** İrfan Uzun, Enes Colak, Zeliha Atlıhan, Çağrı Mutaf, Ali Hakim Reyhan, Funda Yüksekyayla

**Affiliations:** 1 Department of Ophthalmology, Faculty of Medicine, Harran University, 63100 Sanlıurfa, Türkiye; enescolak@harran.edu.tr (E.C.); cagrimutaf@harran.edu.tr (Ç.M.); alihakimreyhan@harran.edu.tr (A.H.R.); f.dilmen@harran.edu.tr (F.Y.); 2 Department of Pediatry, Faculty of Medicine, Harran University, 63100 Sanlıurfa, Türkiye; zelihaatlihan@harran.edu.tr

**Keywords:** childhood obesity, intraocular pressure, central corneal thickness, anterior chamber parameters, specular microscopy, pediatric ophthalmology

## Abstract

**Background/Objectives**: Childhood obesity is a significant health concern also capable of impacting ocular health. This study evaluates the effects of childhood obesity on corneal morphology, anterior chamber parameters, intraocular pressure (IOP), and corneal endothelial cell morphology. Understanding these relationships may contribute to early diagnosis and management strategies. **Methods:** This prospective, cross-sectional study was conducted at the Harran University Faculty of Medicine between January and December, 2024. Ninety children aged 7–17 years were included, with only the right eyes being analyzed. The participants were categorized into three groups based on body mass index (BMI) percentiles: normal weight (≤85th percentile), overweight (86–94th percentiles), and obese (≥95th percentile). All participants underwent comprehensive ophthalmological examinations, including IOP measurement with a non-contact tonometer, corneal topography assessment using a Scheimpflug camera, and endothelial cell morphology evaluation via specular microscopy. **Results**: IOP was significantly higher in the overweight and obese groups (*p* < 0.001). Central corneal thickness (CCT) also increased significantly in these groups (*p* < 0.05). Positive correlations were determined between BMI and IOP (r = 0.493, *p* < 0.001) and CCT (r = 0.345, *p* < 0.001). Additionally, waist circumference exhibited a strong correlation with BMI (r = 0.905, *p* < 0.001) and a significant association with IOP (r = 0.463, *p* < 0.001). No significant differences were observed among the groups in terms of other anterior chamber or endothelial parameters. **Conclusions**: Childhood obesity is associated with increased IOP and CCT, suggesting potential alterations in corneal biomechanics and ocular physiology. These findings highlight the importance of routine ophthalmological evaluation in obese children to detect early ocular changes and prevent long-term complications.

## 1. Introduction

Childhood obesity has become a major global public health problem [1,2]. Obesity is not only associated with chronic conditions such as cardiovascular disease, type 2 diabetes, and some types of cancer, but can also affects ocular health [3,4,5,6]. It creates a state of low-grade inflammation, which can affect various structures of the eye and lead to changes in important ocular parameters, such as corneal morphology, anterior chamber parameters, intraocular pressure (IOP), and refraction [5,6,7,8,9,10]. Studies have shown higher IOP in obese children, and this may exacerbate the risk of glaucoma. The rise in IOP has been attributed either to increased adipose tissue in the orbit due to obesity raising episcleral venous pressure, or to autonomic dysfunction disorders associated with insulin resistance in obese children [3,6]. Central corneal thickness (CCT) is particularly relevant in glaucoma risk assessment, as thicker corneas can lead to an overestimation of IOP. Given that CCT influences IOP measurements, accurate glaucoma risk assessment requires adjusting IOP values according to CCT data [11,12]. Ethnicity, gender and refractive errors may have an effect on CCT and IOP. Significant differences in CCT and IOP values have been observed between different ethnic groups [13,14,15]. For example, individuals of African descent have thinner corneas and higher IOP compared to Hispanics and Whites [15]. However, there are inconsistent results in the literature regarding the effect of gender and refractive errors on CCT and IOP [13,15,16,17]. The body mass index (BMI) is widely used to assess obesity and has been associated with various health problems in adults [18,19]. However, studies examining the relationship between BMI and CCT have yielded conflicting results. While some have reported higher CCT in morbidly obese individuals [20,21], others have detected no significant relationship between obesity and CCT [10,22]. These discrepancies may be due to differences in study populations, methodological variations, and the potential effects of other factors associated with obesity (such as diabetes and sleep apnea) [5,10].

The effect of obesity on corneal morphology and biomechanics is not yet fully understood. Although there are studies suggesting a relationship between obesity and increased corneal steepness [23,24], there are also studies reporting no significant change in keratometric values [21,25]. However, some studies have suggested an association between obesity and corneal stiffness [26]. The number of studies evaluating the effect of obesity on corneal endothelial cell morphology in children is limited. Kurtul et al. reported that obesity had no significant effect on endothelial cell morphology [5]. 

This study evaluated the relationship between BMI, waist circumference (WC), corneal morphology, corneal endothelial morphology, anterior chamber parameters, and IOP in childhood. The findings will yield more information about the potential effects of obesity on ocular health and provide a basis for early diagnosis and treatment strategies.

## 2. Materials and Methods

This prospective cross-sectional study was performed at the Harran University Medical Faculty ophthalmology and pediatric clinics, Türkiye, between January and December, 2024. It was conducted in accordance with the principles of the Declaration of Helsinki and was approved by the Harran University ethical committee (04.01.2024-296319). Ninety right eyes of 90 children aged between 7 and 17 years were included in the study. Individuals not using contact lenses, with no optical problems other than refractive error, with no history of surgery, with no ocular pathology, and with no ophthalmological pathology determined following examination and tests, were enrolled.

All cases included in the study underwent detailed ophthalmological evaluations including slit-lamp examination, IOP measurement, visual acuity, and dilated fundus examinations. IOP was measured using a NIDEK NT-2000 non-contact tonometer (Nidek, Tokyo, Japan) by taking three consecutive readings and calculating the mean value, with measurements performed between 09:30 and 11:00. Corneal topography and anterior segment parameters were assessed using a Pentacam Scheimpflug topography device (Pentacam, Wetzlar, Germany), measuring K flat, K steep, Kmax, CCT, corneal volume (CV), anterior chamber volume (ACV), anterior chamber depth (ACD), and anterior chamber angle (ACA). Endothelial cell morphology was evaluated with a NIDEK CEM-530 specular microscope (Nidek, Tokyo, Japan), analyzing at least 110 cells per measurement. The endothelial parameters assessed included endothelial cell density (ECD), hexagonality (HEX), and coefficient of variation (CV). All measurements were performed by the same trained technician. BMI was calculated by dividing the patient’s weight by the square of the height (kg/m^2^). Children were categorized as Group 1 (normal-weight; BMI ≤ 85), Group 2 (overweight; BMI 86–94), and Group 3 (obese; BMI ≥ 95), according to Turkish percentile reference values (Olcay Neyzi percentile calculation system) [27]. The 90 participants (47 boys, 43 girls) were stratified into three BMI-based cohorts: normal weight (*n* = 30), overweight (*n* = 30), and obese (*n* = 30). Mean ages were 11.3 ± 2.56 years in the normal weight group, 11.7 ± 3.14 in the overweight group, and 11.6 ± 3.40 in the obese group. WC was calculated using a measuring tape, with the measurement rounded to the nearest half-centimeter halfway between the iliac crest and the lower rib margin.

Statistical analysis was performed on Statistical Package for the Social Sciences version 25 software. The Kolmogorov–Smirnov test was applied to assess the assumption of normality of numerical variables. Continuous variables were expressed as mean ± standard deviation and compared using the independent samples *t* test. One-Way ANOVA (Welch’s test) was used for variables with normal distributions when comparing more than two groups. The post hoc Tukey test was applied for variables exhibiting significant differences. Pearson correlations were used to assess relationships between continuous variables. The chi-square test was applied to compare categorical variables, expressed as numbers and percentages (%). A *p* value of less than 0.05 was regarded as statistically significant.

## 3. Results

Mean ages were similar across groups with no significant differences (*p* > 0.05). There was also no statistically significant gender difference between the groups (*p* > 0.05). As expected, BMI and WC values were significantly higher in the overweight and obese groups compared to the normal-weight individuals (*p* < 0.001). Tukey post hoc analysis further confirmed these findings, revealing significant differences in BMI between normal-weight and overweight individuals (−8.64, *p* < 0.001), normal-weight and obese individuals (−13.06, *p* < 0.001), and overweight and obese individuals (−4.43, *p* < 0.001). Similarly, WC differed significantly between normal-weight and overweight individuals (−37.4, *p* < 0.001) as well as normal-weight and obese individuals (−45.10, *p* < 0.001), while the difference between overweight and obese groups only approached significance (−7.73, *p* = 0.056). Additionally, IOP was significantly elevated in the overweight and obese groups compared to the normal-weight cohort (*p* < 0.001). Post hoc analysis confirmed these differences, showing a significant increase in IOP between normal-weight and overweight individuals (−2.17, *p* < 0.001) and between normal-weight and obese individuals (−2.90, *p* < 0.001). However, the difference between overweight and obese groups (−0.733, *p* = 0.355) was not statistically significant. This suggests a relationship between increased BMI and altered ocular dynamics, potentially positioning obesity as a risk factor for elevated IOP in pediatric populations. Table 1 summarizes the general characteristics of the study groups. 

CCT was significantly greater in overweight and obese individuals (*p* < 0.05), suggesting potential biomechanical changes associated with increased body mass. Tukey post hoc analysis revealed significant mean differences between the normal-weight and overweight (−12.0, *p* = 0.060), normal-weight and obese (−23.6, *p* < 0.001), and overweight and obese groups (−11.6, *p* = 0.073). The most pronounced difference was observed between normal-weight and obese individuals. CV, keratometric parameters (K flat, K steep, Kmax), and anterior chamber parameters (ACV, ACD, and ACA) exhibited no significant intergroup differences (*p* > 0.05), suggesting that while corneal thickness may be BMI-dependent, other anterior segment metrics remain stable across weight categories. Table 2 shows a comparison of the mean corneal values and anterior segment parameters in the study group.

No significant differences were observed in corneal endothelial parameters between the normal, overweight, and obese groups (*p* > 0.05). ECD and CV also remained comparable across all three groups. Although HEX exhibited a slight decrease in overweight and obese individuals, the difference was not statistically significant. Table 3 shows a comparison of the mean corneal endothelial parameter values in the study group.

**Figure 1:**
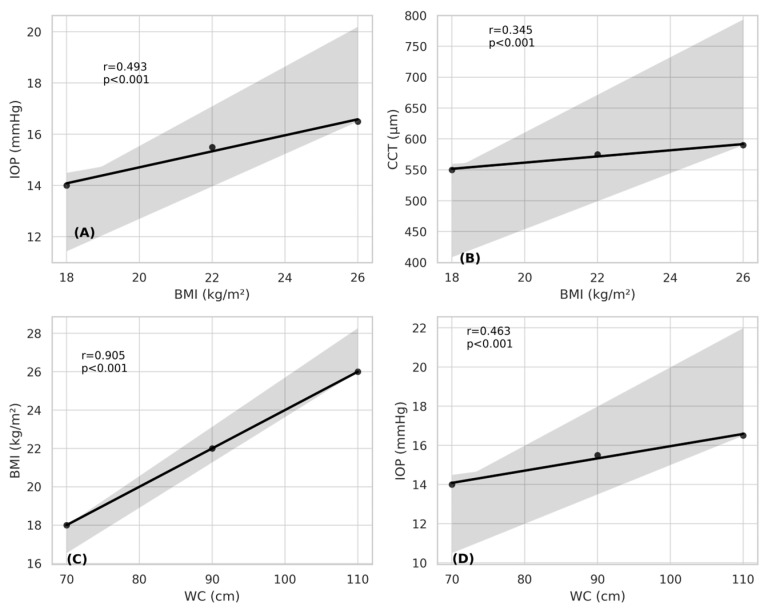
Scatter plots illustrating Pearson correlation coefficients for mean values. (**A**) Correlation between BMI and IOP; (**B**) correlation between BMI and CCT; (**C**) correlation between WC and BMI; (**D**) correlation between WC and IOP. CCT, central corneal thickness; WC, waist circumference; IOP, intraocular pressure.

A strong positive correlation was observed between BMI and IOP (r = 0.493, *p* < 0.001), supporting the hypothesis that increased BMI contributes to elevated IOP. A significant correlation was also identified between BMI and CCT (r = 0.345, *p* < 0.001), suggesting that BMI affects corneal structural properties. WC exhibited a robust correlation with BMI (r = 0.905, *p* < 0.001) and a significant association with IOP (r = 0.463, *p* < 0.001), further confirming the systemic interplay between obesity and ocular physiology (Figure 1). However, BMI was not significantly correlated with either keratometric parameters or anterior chamber indices (*p* > 0.05), implying that corneal curvature and anterior segment architecture remain unaffected by variations in body mass.

## 4. Discussion

This study investigated the impact of pediatric obesity on ocular health by assessing associations between BMI, WC, corneal morphology, corneal endothelial morphology, anterior chamber characteristics, and IOP. The growing prevalence of childhood obesity underscores the urgent need to understand its ophthalmic consequences [28]. Early identification of ocular abnormalities and timely intervention are of paramount importance in mitigating long-term visual impairment. This study, involving 90 children aged 7–17 years, provides a detailed analysis of obesity-related ocular changes and their potential pathophysiological underpinnings, providing a foundation for future investigative and clinical activities.

A key finding of this study was that IOP was significantly higher in overweight and obese children compared to the normal weight group. Tukey post hoc analysis confirmed a statistically significant increase in IOP in the overweight and obese groups relative to the normal-weight group. These findings support the hypothesis that obesity may contribute to an elevated risk of glaucoma by increasing IOP during childhood. The positive correlations of BMI and WC with IOP suggest the presence of systemic effects of obesity on ocular physiology. These findings are consistent with previous studies reporting a relationship between childhood obesity and increased IOP [29,30,31,32,33,34]. However, other studies have reported that obesity has no significant effect on IOP [5,22]. This inconsistency may be attributable to methodological differences and heterogeneous study populations. The mechanisms underlying the relationship between obesity and elevated IOP remain unclear, although various hypotheses have been proposed. Possible mechanisms underlying IOP elevation in obesity include increased episcleral venous pressure due to excess adipose tissue and autonomic dysfunction linked to insulin resistance in obese children [4,22,29,35,36,37]. IOP represents a significant risk factor for the development of glaucoma [4,37]. These findings emphasize the need for regular IOP monitoring in overweight and obese children, particularly in those with a family history of glaucoma. Monitoring should be conducted at least annually, with additional assessments being recommended for individuals exhibiting progressive IOP elevation. Specific screening methods, such as Goldmann applanation tonometry and optical coherence tomography for retinal nerve fiber layer analysis, should be prioritized to enhance early detection and risk stratification.

Significantly greater CCT was also observed in overweight and obese participants compared to those with normal weight. Tukey post hoc analysis revealed the most pronounced differences in CCT between the normal-weight and obese groups. This suggests that increased body mass may be associated with biomechanical alterations in the cornea. Previous research into the relationship between BMI and CCT has yielded conflicting results. Some studies reported increased CCT in morbidly obese individuals [20,21,38], whereas others have established no significant association between obesity and CCT [10,22]. Some differences have been observed between animal models and human studies. Although studies in mice have not shown a direct relationship between CCT and obesity, obesity has been shown to cause corneal dysfunction, and these changes appear to precede hyperglycemia. This finding suggests that corneal biomechanical changes begin early, and that similar effects in the human population should be investigated more extensively. Furthermore, a high-fat diet has been found to have long-term adverse effects on corneal health that cannot be fully reversed by dietary modification [7]. This suggests that the corneal changes associated with obesity may be potentially permanent, and longitudinal studies in humans are needed. Given the methodological differences, heterogeneity of study populations and the effects of comorbidities, these results should be carefully evaluated both in terms of animal models and clinical trials. The observed increase in CCT in obese children has critical implications for glaucoma risk assessment and refractive surgery planning, necessitating refinements in screening protocols.

While the findings of this study confirm significant CCT elevation in overweight and obese children, the exact mechanisms underlying this relationship remain unclear. Several hypotheses have been proposed, including systemic inflammation, increased corneal hydration due to metabolic dysregulation, and collagen remodeling associated with obesity-related biochemical alterations [5,7,10,20,21,38]. Longitudinal studies are now needed to determine whether these corneal changes progress over time and whether weight reduction strategies can reverse or mitigate these effects. 

A number of studies have suggested that corneal hysteresis (CH) and corneal resistance factor (CRF), both of which provide insights into corneal biomechanical properties, may also be affected by obesity [3,26,32]. Furthermore, CH and CRF have been shown to influence IOP measurement and are associated with glaucoma types or severity of glaucoma [39]. CCT also significantly affects IOP measurements, as thicker corneas can lead to falsely high measurements; therefore, accounting for CCT variations is essential for accurate glaucoma risk assessment [11,12]. These biomechanical changes can influence clinical decision-making, particularly in refractive surgery, in which corneal stability is crucial, and in glaucoma risk assessment, since corneal properties affect the accuracy of IOP measurements. Understanding these variations can be useful in refining screening and management strategies for obese patients. Increased corneal stiffness in obese individuals may have implications for refractive surgery outcomes and glaucoma risk assessment, since corneal biomechanical properties affect the accuracy of IOP measurement.

In contrast, no significant differences were identified in CV, keratometric parameters (K flat, K steep, and Kmax), or anterior chamber parameters (ACV, ACD, and ACA) among the groups. These findings indicate that while corneal thickness is affected by BMI, other anterior segment parameters remain largely unchanged by variations in body mass. The lack of an association between BMI and these anterior segment metrics suggests that obesity does not necessarily compromise overall corneal shape and structure beyond thickness-related changes.

Research into the effect of obesity on corneal morphology is currently limited. In contrast to previous studies suggesting a correlation between obesity and increased corneal steepness, the results of the present study do not support this association. While some earlier studies have linked obesity to increased corneal steepening and potential keratoconus susceptibility [23,24], the current findings indicate no significant differences in keratometric values across BMI groups. Similarly to the present study, Dogan et al. observed no significant changes in keratometric values or anterior chamber parameters [20]. This discrepancy may stem from variations in study designs, including differences in age groups, measurement techniques, and sample sizes. Genetic predisposition, regional metabolic effects, and biomechanical variations in the cornea may also have contributed to these inconsistencies. In the light of the conflicting results in the current literature, longitudinal studies with larger cohorts are now needed to determine whether obesity exhibits a delayed impact on corneal biomechanics.

The study findings revealed no significant differences in corneal endothelial parameters among normal, overweight, and obese individuals. ECD, CV, and HEX also remained comparable across the groups, suggesting that increased BMI values do not significantly impact corneal endothelial morphology. These findings align with those of a previous study indicating that variations in BMI do not lead to endothelial dysfunction [5]. However, the long-term effects of obesity on corneal health remain unclear, and further research with larger sample sizes and longer follow-up periods is now needed to assess potential subclinical changes. Additionally, factors such as metabolic syndrome and systemic inflammation, which are more prevalent in cases of obesity, may contribute to subtle alterations in corneal physiology that were not detected in this study.

Kurtul et al. [5] reported significantly greater foveal retinal thickness, choriocapillaris flow area, and superficial and deep foveal capillaries in obese children compared to healthy controls. This suggests that obesity may affect retinal microvasculature. Such findings appear to indicate that obesity may heighten the risk of macular pathology. Choroidal and retinal thinning may be associated with systemic vascular dysfunction, oxidative stress, and inflammatory processes that are widely prevalent in obesity. These changes may have long-term consequences, including an increased susceptibility to age-related macular degeneration and other retinal disorders [5].

The results of this study yield critically important insights into the ocular implications of childhood obesity. The observed increases in IOP and CCT underscore the need for routine ophthalmic monitoring in obese children to facilitate the early detection of potential glaucoma risks. Furthermore, since childhood obesity is frequently associated with metabolic syndrome, systemic inflammation, and vascular dysregulation, these factors may contribute to ocular changes that require further investigation. Routine screening of obese children should include comprehensive eye exams that assess IOP, CCT, and retinal parameters.

Preventive strategies should include lifestyle modifications, such as promoting a balanced diet and regular physical activity, early screening for metabolic disorders through routine blood glucose and lipid profile testing, and ophthalmological follow-ups with periodic IOP measurements and retinal imaging. These measures are particularly important for children with additional risk factors, such as a family history of glaucoma or diabetes. Due to the rising prevalence of pediatric obesity, interdisciplinary collaboration between pediatricians and ophthalmologists is essential for a holistic approach to preventing obesity-related ocular complications.

This study has several limitations. First, the sample size was relatively modest, which may have implications for the generalizability of the findings. In addition, due to its cross-sectional nature, it was not possible to determine the causal relationship between obesity and ocular parameters. Furthermore, the findings may not be generalizable to other ethnic populations, since only Turkish children were investigated.

Future prospective studies should include larger sample sizes and longitudinal methodologies to assess the long-term ophthalmic effects of pediatric obesity and investigate the contributions of comorbid conditions such as insulin resistance and sleep apnea to obesity-related ocular changes. Advanced imaging techniques, especially optical coherence tomography angiography, will be crucial to reveal subtle microvascular changes in retinal and choroidal tissues. Research should prioritize the investigation of the underlying molecular and cellular mechanisms linking CCT changes to obesity, specifically examining how adipokines, inflammatory cytokines and metabolic dysregulation affect corneal architecture and biomechanical integrity. Well-designed human studies with standardized protocols and comprehensive physiological assessments are essential to clarify the temporal progression of these changes and determine whether obesity-induced ocular manifestations can be reversed by various interventions, ultimately informing more effective preventive and therapeutic strategies.

## 5. Conclusions

This study highlights the potential ocular ramifications of childhood obesity, particularly in terms of increased IOP and CCT. These findings have important implications for assessing ophthalmic risks in obese children and implementing appropriate preventative strategies. Regular monitoring and early intervention may mitigate the risk of future ocular complications associated with obesity. A deeper understanding of the intersection between obesity and ocular health may ultimately contribute to more effective clinical interventions aimed at mitigating obesity-related ocular complications.

In summary, a comprehensive approach to pediatric obesity should incorporate ophthalmic screening to ensure early detection of potential vision-threatening conditions. Addressing obesity from both systemic and ocular perspectives will permit more targeted healthcare strategies that prioritize long-term visual and overall health outcomes.

## Figures and Tables

**Table 1:** Characteristics of the study groups.

	Normal (n = 30)	Overweight (n = 30) *p*	Obese (n = 30) *p*	*p* ^c^
Age (years)	11.3 ± 2.56	11.7 ± 3.14 0.530 ^a^	11.6 ± 3.40 0.639 ^a^	0.797
Gender Male/Female-n (%)	14 (46.7)/16 (53.3)	14 (46.7)/16 (53.3) 1 ^b^	12 (40)/18 (60) 0.610 ^b^	0.840
BMI (kg/m^2^)	18.6 ±1.98	23.3 ± 1.92 **<0.001** ^a^	27.2 ± 1.93 **<0.001** ^a^	**<0.001**
WC (cm)	66 ± 9.55	103 ± 16.7 **<0.001** ^a^	111 ± 11 **<0.001** ^a^	**<0.001**
IOP (mmHg)	13.6 ± 1.52	15.8 ± 2.35 **<0.001** ^a^	16.5 ± 2.19 **<0.001** ^a^	**<0.001**

Mean ± standard deviation. Values with statistical significance are shown in bold. ^a^ Comparison with the controls (independent samples *t* test). ^b^ Comparison with the controls (chi-square test). ^c^ Comparison within the groups (One-Way ANOVA, Welch’s test). A *p* value less than 0.05 was considered statistically significant. WC, waist circumference; IOP, intraocular pressure.

**Table 2:** A comparison of mean corneal values and anterior segment parameters.

	Normal (n = 30)	Overweight (n = 30) *p* ^a^	Obese (n = 30) *p* ^a^	*p* ^b^
CCT (μm)	537 ± 23.9	549 ± 21.7 **0.046**	561 ± 22.5 **<0.001**	**<0.001**
K flat (D)	42.4 ± 0.808	42.3 ± 1.33 0.652	42.2 ± 1.01 0.763	0.751
K steep (D)	43 ± 0.808	43.4 ± 1.49 0.724	43.1 ± 1.17 0.563	0.445
Kmax (D)	42.8 ± 0.837	42.9 ± 1.39 0.263	42.7 ± 1.06 0.283	0.722
CV (mm^3^)	59.1 ± 2.32	59.3 ± 3.59 0.779	59.5 ± 2.68 0.310	0.833
ACV (mm^3^)	199 ± 29.9	201 ± 28.2 0.218	203 ± 29 0.622	0.201
ACD (mm)	3.13 ± 0.254	3.14 ± 0.238 0.896	3.20 ± 0.267 0.376	0.544
ACA (°)	39.8 ± 5.10	40.1 ± 5.26 0.830	40.2 ± 5.37 0.765	0.186

Mean ± standard deviation. Values with statistical significance are shown in bold. ^a^ Comparison with the controls (independent samples *t* test). ^b^ Comparison within the groups (One-Way ANOVA, Welch’s test). A *p* value less than 0.05 was considered statistically significant. CCT, central corneal thickness; CV, corneal volume; ACV, anterior chamber volume; ACD, anterior chamber depth; ACA, anterior chamber angle; max, maximum.

**Table 3:** A comparison of mean corneal endothelial parameter values in the study groups.

	Normal (n = 30)	Overweight (n = 30) *p* ^a^	Obese (n = 30) *p* ^a^	*p* ^b^
ECD (cells/mm^2^)	3128 ± 257	3123 ± 270 0.753	3121 ± 265 0.664	0.903
CV	28.6 ± 3.54	28.5 ± 4.52 0.919	28.2 ± 4.71 0.591	0.852
HEX (%)	69.7 ± 9.91	67.8 ± 10.15 0.106	67.4 ± 10.61 0.074	0.162

Mean ± standard deviation. ^a^ Comparison with the controls (independent samples *t* test). ^b^ Comparison within the groups (One-Way ANOVA, Welch’s test). A *p* value less than 0.05 was considered statistically significant. ECD, endothelial cell density; CV, coefficient of variation; HEX, hexagonal cell ratio.

## Data Availability

The data presented in this study are available from the corresponding author upon request. The data are not publicly available, due to ethical restrictions.
